# Toward liveable commercial streets: A case study of Al-Karada inner street in Baghdad

**DOI:** 10.1016/j.heliyon.2019.e01652

**Published:** 2019-05-20

**Authors:** Noor Mazin Ghazi, Zaynab Radi Abaas

**Affiliations:** a Ministry of Construction, Housing and Public Municipalities, Engineering Consultancy Center, 10001, Baghdad, Iraq; b University of Baghdad, College of Engineering, Architecture Department, Baghdad, Iraq

**Keywords:** Architecture

## Abstract

Nowadays, liveability is a major consideration in upgrading public spaces, especially more sustainable streets. Considerable research has also been conducted to determine the most relevant indicators for more vibrant commercial streets. This paper sheds light on a specific case study in which terrorist attack led to the loss of vitality and ability to attract visitors in one of the most important commercial areas in Baghdad, Al-Karada inner street. The paper's aim is to identify indicators for reviving the companionable and sociable life of a street. As a result, it presents many indicators that can be applied to upgrade commercial street vitality by way of a significant, inclusive checklist, which incorporates many aspects. This checklist serves as a comprehensive framework to present needed information, diagnose problems, and identify the strengths and weaknesses of a commercial street's performance to enhance its liveability.

## 1 Introduction

Streets are the spaces in which cities breathe. As Whyte notes, ‘The road is the stream of life of the city, where we meet up, the pathway to the core’ [1]. Since ancient times, the liveable street has been a place for people not only to cross or walk, but also to engage in commercial, social, and recreational activities. By the late 1950s, two conflicting approaches to street development were proposed. First was to increase the capacity of the streets to accommodate rapidly expanding populations and vehicles. Second was to provide diverse streets able to accept various public transport options and a pedestrian network instead of cars [2, 3, 4]. Currently, the global trend is towards vibrant streets that meet needs ranging from food and basic safety to beauty, cultural impression, and sense of belonging to community or place, encouraging people to stay longer and thus improving quality of life in public spaces [5, 6]. Moreover, studies have revealed several key aspects for upgrading the liveability of streets, each with measurable sub-indicators. First is the design and location aspect, which relates to the street's location and spatial characteristics [7, 8]. Second is the social and cultural aspect [8, 9, 10], and third is the urban planning aspect [7, 11]. This paper aims to introduce indicators and sub-indicators within these key aspects for streets, and to develop a checklist scale to help architects and planners in Iraq evaluate commercial streets and improve their liveability. Additionally, a field study was conducted to apply the proposed checklist and identify the issues that prevent such streets from becoming vibrant and full of life. The case study addresses one of the most notable urban areas in Baghdad, Al-Karada inner street, which prior to terrorist bombing in 2016 was a primary location for shopping and gathering. The limitation of this research is formed by finding a liveability checklist from reviewing the most relevant theories (Jane Jacobs, Jan Gehl, Donald Applyard, William Whyte, etc.) which listed in the literature and sources, then applying it to AL- Karrada inner Street as a preliminary test.

The paper is structured as follows: Section one introduces the key aspects and main indicators of liveable streets, ending with a summary of the developed checklist. Section two applies the finalized framework to the selected case study area in Baghdad, explaining the location and field measurements. Section three presents discussion and evaluation. Finally, section four reports the results and a summary of the case study, ending with final conclusions.

## 2 Theory

### 2.1 Theories of liveability (aspects and indicators)

Numerous books, studies, and papers have demonstrated the importance of the key aspects of design and location, society and culture, and urban planning for upgrading the liveability of public streets [6, 12, 13] The design and location aspect focuses on physical, design, and spatial improvements, and incorporates many features, beginning with a street's nature and its domination by either vehicles or pedestrians, and reaching to the edges of the street, street furniture, and design details [14, 15]. The social and cultural aspect concerns the suitability of the street for people and its ability to meet their social demands; it must reflect the community's identity through collective memory and place context [10, 16, 17]. Finally, the urban planning aspect takes a bottom-up approach concentrating on street accessibility, density, diversity, and context in a human scale.

#### 2.1.1 Design and location aspect

**Fig. 1:**
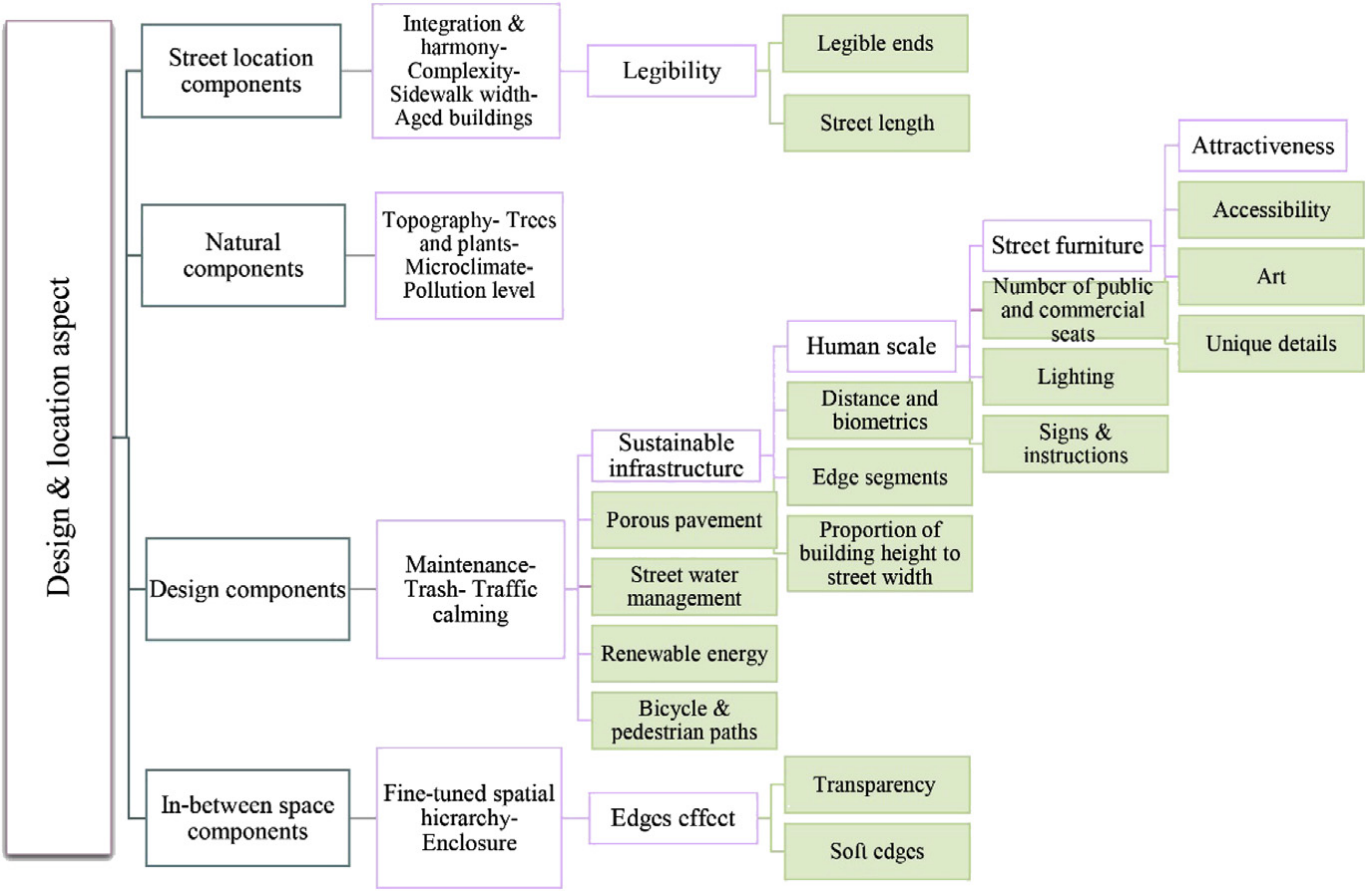
Four main indicators in the design and location aspect: In-between space components, street location components, natural components, and design components. Each has multiple sub-indicators. Source: author.

Many researchers have explained the flexibility of the design and location aspect for accumulative indicators. For main components can be considered: in-between space components, street location components, natural components, and design components. In-between space components, which are particularly crucial, relate to edges, details, and facades between indoor and outdoor spaces [7, 18, 19]. Alexander, Ishikawa, and Silverstein (1977) clarified in their book that ‘If the edge fails, then the space never becomes lively’ [20]. Additionally, these components should demonstrate a hierarchical transition from public to semi-public–semi-private and private, moving from the outside to the inside of the building, with effective edges that facilitate greater intimacy and safety. Street location components include many variables, for example the ability of the street and sidewalk to accommodate diverse activities [17, 21, 22, 23], the diversity of building patterns providing more user opportunities [24], and the length and distinct ends of streets [25]. Natural components, represented by trees and plants, have an important role in protecting the street from weather conditions, drawing street barriers and topographies, and defining the street scene [22, 26, 27, 28]. Finally, design components and their variables include the technical elements of the street that can be seen, interactive calls for motor activities or dialogue as an integral part of the built environment or independent and stand-alone [29], providing attractive, comfortable, and safe walkways and bicycle lanes; reducing congestion; maintaining public health; and reducing environmental pollution, noise, and visual impairment. The ultimate goals of such elements are definition of the street space, containment, maintenance, humanitarianism, calmness, comfortableness, and efficiency [30]. As Allan Jacobs (1995) cited in his research, ‘It's no big mystery. The best streets are comfortable to walk along with leisure and safety. They are streets for both pedestrians and drivers. They have definition, a sense of enclosure with their buildings; distinct ends and beginnings, usually with trees. Trees, while not required, can do more than anything else and provide the biggest bang for the buck if you do them right. The key point again, is great streets are where pedestrians and drivers get along together’ [31]. Fig. 1 illustrates the collective indicators of the design and location aspect.

#### 2.1.2 Social and cultural aspect

This aspect concerns social life and communication in the street, considering that streets are places with social value, not merely physical spaces. Two indicators comprise this aspect. The first involves social components — personalization, vending and kiosks, shows and performances, activities, and human desires and needs — which can be taken into account when upgrading a street to achieve a more cohesive society. The second involves cultural components, which include sense of place, taking into consideration local and regional contexts; the time factor; adequate personal space; and demographic structure.

**Fig. 2:**
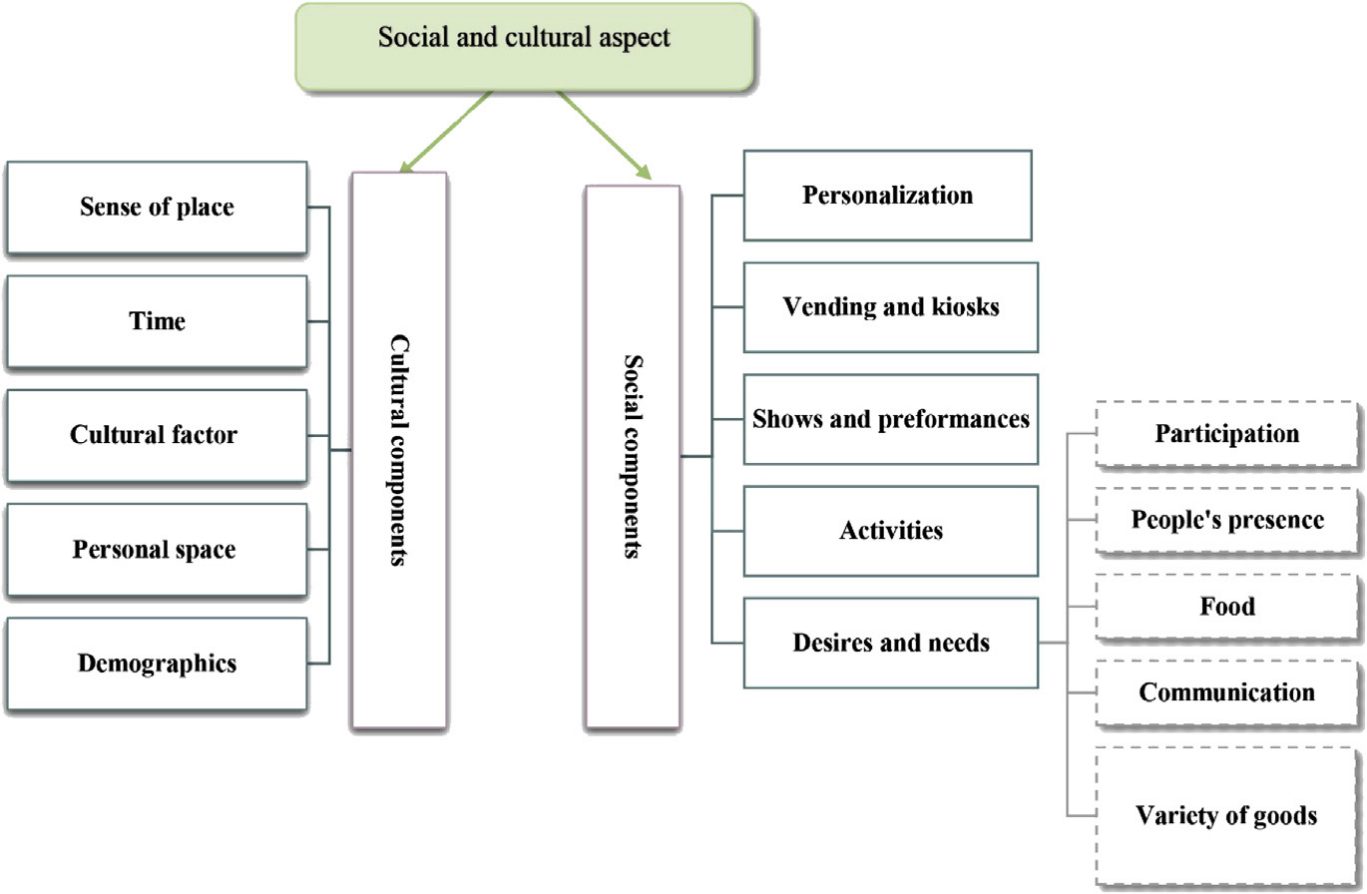
The social and cultural aspect contains two main indicators, social components and cultural components. Social components include personalization, vending and kiosks, shows and performances, activities, and human desires and needs. Cultural components include sense of place, time, cultural factors, personal space, and demographics. Source: author.

Exclusively public space should represent an entirely open place for communication and safety [32]. When undesirable street vendors and performers attract more visitors, they compete daily for adequate space. Many policies have attempted to solve these conflicts [22]. In addition, types of activities that take place in the street are classified as necessary, optional, and social activities, each with its own nature and visitors, and each simulating the others to bring more visitors and activities [33, 34]. In addition to the nature of exhibits and the use of lighting, signs and colours can attract or repel people. All this leads to greater social participation, gathering, and talking, which in turn provides greater linkage [19, 35, 36]. As Jan Gehl notes, ‘A good city is like a good party – people stay for much longer than really necessary, because they are enjoying themselves’ [37]. With respect to enhancing the success of the urban street, Gehl stressed that people communicate through three processes (hearing, vision, and talking), and that communication is affected by the level of pedestrian density and the length of fixed events, including social events and communication [38]. Providing convenient transportation and good accessibility activates more liveable streets [17, 39], as do, as noted by Allan Jacobs, ‘good food, good service, good company’ [25]. In contrast, cultural components give streets a sense of place and a place identity that considers the local and regional context [17]. They also take into account diversity, change, and a sense of history from the street's years and the successive decisions made regarding design, construction, or reconstruction [25]. To understand these differences, Hall divided societies into two parts [40]: high context cultures and low context cultures. Each has private and personal characteristics for identifying its comfort position in the street [41] (see Fig. 2).

#### 2.1.3 Urban planning aspect

**Fig. 3:**
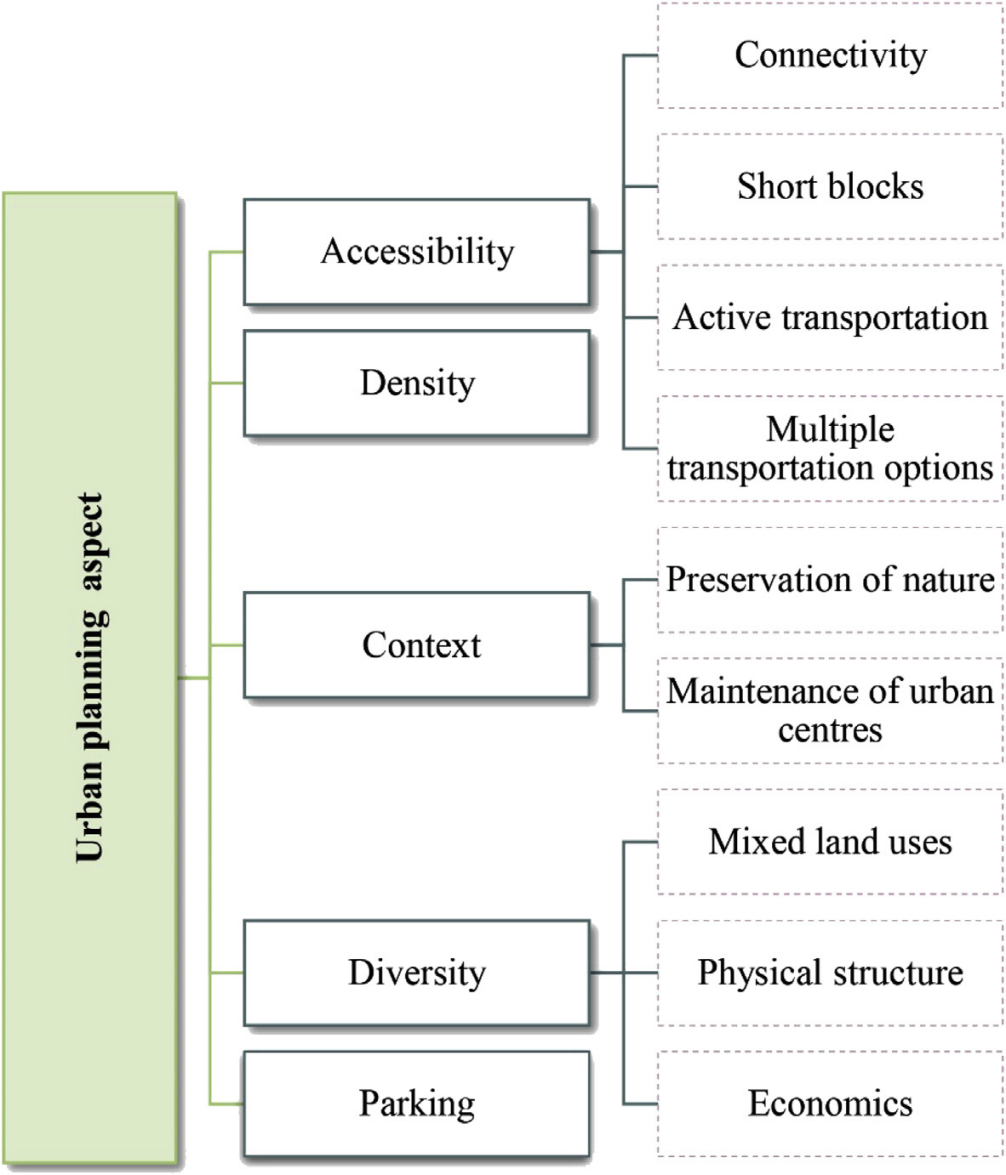
The five main indicators of the urban planning aspect with their relative sub-indicators. Source: author.

Research has pointed to many indicators at the urban planning level, such as accessibility, density, context, diversity, and adequate parking space. Accessibility as an indicator can be characterized by four sub-indicators: connectivity, short blocks, active and sustainable transportation, and multiple transportation options. Connectivity refers to the ability to reach the area from many directions, or to easily access goods, services, activities, and destinations, called ‘opportunities’ [42, 43]. Jacobs (1961) effectively illustrated the importance of short blocks: ‘frequent streets and short blocks are valuable because of the fabric of intricate cross-use that they permit among the users of a city neighbourhood’ [24, 44]. The second main indicator is high density among people, which promotes social and economic exchange, thus facilitating movement and closeness in the community [45]. Context relates both to fitting in with the larger context, and supporting the existing local context [12, 46]. It also indicates conserving natural elements, open spaces, and plantations, and preserving urban centres [47]. The diversity indicator refers to variety in physical structures, economic systems, social composition, land uses, building patterns, and urbanization to meet the actual needs of the population [25, 48]. It is key to building the economy; convergence alone is not sufficient [45]. As Jacobs (1961) referred to in her book on the life and death of great American cities, ‘The district, and indeed as many of its internal parts as possible, must serve more than one primary function; preferably more than two. These must ensure that presence of people who go outdoors on different schedules and are in the place for different purposes, but who are able to use many facilities in common’ [24]. Finally, parking is a key factor in the success of the urban aspect [49]. All indicators of the urban planning aspect and their relative sub-indicators are illustrated in Fig. 3.

### 2.2 Model of liveability(evaluating checklist)

The theoretical review conducted in this research provided an extensive study of the most important theories and literature concerning the concept of liveability as a key factor in upgrading commercial streets, resulting in a comprehensive knowledge base regarding the most important indicators for raising the efficiency of street performance and increasing street liveability. Based on the aspects identified and their indicators, a checklist was developed including the most important indicators of the design and location aspect, the social and cultural aspect, and the urban planning aspect (Table 1). This checklist was then applied to Al-Karada inner street in Baghdad, an important liveable site that lost important attractive factors due to external circumstances (see Table 2).

**Table 1:** Proposed checklist for evaluating liveability, designed and developed by the researchers.

Design and location aspect					Social and cultural aspect				
Design components					Social components				
Indicators		Good	Neutral	Bad	Indicators		Good	Neutral	Bad
Attractiveness	Accessibility	☑	☐	☒	Personalization		☑	☐	☒
	Art	☑	☐	☒	Vending & kiosks		☑	☐	☒
	Unique details	☑	☐	☒	Shows and performances		☑	☐	☒
Street furniture	Seating	☑	☐	☒	Activities		☑	☐	☒
	Lighting	☑	☐	☒	Desires and needs	Participation	☑	☐	☒
	Signs	☑	☐	☒		People's presence	☑	☐	☒
Human scale	Distance and biometrics	☑	☐	☒		Food	☑	☐	☒
	Edge segments	☑	☐	☒		Communication	☑	☐	☒
	Proportion of building height to street width	☑	☐	☒		Variety of goods	☑	☐	☒
Sustainable infrastructure	Porous pavement	☑	☐	☒	Cultural components				
	Street water management	☑	☐	☒	Sense of place		☑	☐	☒
	Renewable energy	☑	☐	☒	Time		☑	☐	☒
	Bicycle and pedestrian paths	☑	☐	☒	Cultural factor		☑	☐	☒
Traffic calming		☑	☐	☒	Personal space		☑	☐	☒
Trash		☑	☐	☒	Demographics		☑	☐	☒
Maintenance		☑	☐	☒	Urban planning aspect				
Natural components					Indicators		Good	Neutral	Bad
Topography		☑	☐	☒	Accessibility	Connectivity	☑	☐	☒
Trees and plants		☑	☐	☒		Short blocks	☑	☐	☒
Microclimate		☑	☐	☒		Active transportation	☑	☐	☒
Pollution level		☑	☐	☒		Multiple transportation options	☑	☐	☒
Street location components					Context	Maintaining natural component	☑	☐	☒
Aged buildings		☑	☐	☒		Maintaining urban centres	☑	☐	☒
Legibi-lity	Legible ends	☑	☐	☒	Diversity	Land use	☑	☐	☒
	Street length	☑	☐	☒		Physical structures	☑	☐	☒
In-between space components						Economics	☑	☐	☒
Enclosure		☑	☐	☒	Density		☑	☐	☒
Fine-tuned spatial hierarchy		☑	☐	☒	Parking		☑	☐	☒
Edges effect	Transparency	☑	☐	☒					
	Soft edges	☑	☐	☒

**Table 2:** Answers to the questionnaire based on the liveability checklist for Al-Karada inner street.

List of indicators for evaluating Al-Karada inner street							
Location and design aspect							
Indicators				Rating			Notes
				Bad	Neutral	Good	
Sub-indicators	Design components	Attract-iveness	Accessibility	37.8	37.8	24.5	
			Art	86.4	10.5	3.1	
			Unique details	93.3	0	6.7	
		Street furniture	Number of public and commercial seats	73.4	18.4	8.2	
			Lighting	54.1	28.6	17.3	
			Signs and instructions	89.7	4.1	6.2	
		Human scale	Distance and biometrics	11.2	41.8	46.9	
			Edge segments	5.1	21.6	73.2	
			Proportion of building height to street width	7.7	34.7	57.6	
		Sustain-able infra-structure	Porous pavement	Non-existent			
			Street water management	60.8	24.4	14.8	
			Renewable energy	Non-existent			
			Bicycle and pedestrian paths	70.2	21.6	8.3	
		Traffic mitigation		11.2	52.1	36.7	
		Trash		85.5	10.2	4.3	
		Maintenance		61.6	20.9	17.6	
	Natural components	Topography		Non-existent			
		Trees and plants		64.3	25.5	10.2	
		Microclimate		74	21.9	41	
		Pollution level		61.3	22.4	16.3	
	Street location components	Sidewalk width		12.8	12.2	75	
		Complexity		Not measured			
		Integration & harmony		72	20.1	7.9	
		Aged buildings		76.2	12.4	11.4	
		Dist-inction	Distinct ends	70.9	16.7	11.6	
			Street length	72.9	22.4	4.7	
	In-between space components	Enclosure		Not measured			
		Fine-tuned spatial hierarchy		39.6	35.4	25	
		Edges effect	Transparency	39.6	35.4	25	
			Soft edges	39.6	35.4	25	
Social and cultural aspect							
Sub-indicators	Social components	Personalization		54.1	24.5	21.4	
		Vending & kiosks		72.6	19.3	8.1	
		Shows & performances		76.1	14.2	9.7	
		Activities		6.4	9	84.6	
		Needs & desires	Participation	26.2	12.2	61.7	
			People's presence	6.2	12.2	81.7	
			Food	20.2	15.9	63.9	
			Communication	31.9	32	36.1	
			Variety of goods	6.4	9	84.8	
	Cultural comp-onents	Sense of place		Not measured			
		Time		Not measured			
		Cultural factors		Not measured			
		Personal space		66.4	25.5	8.1	
		Demographics		13.4	24.7	61.9	
Urban planning aspect							
Sub-indicators	Urban planning aspect	Access-ibility	Connectivity				
			Short blocks				
			Active transportation	69.8	21.9	8.3	
			Multiple transportation options	19.3	24.5	56.1	
		Context	Maintenance of natural components	Non-existent			
			Maintenance of urban centres	24.5	20.4	59.1	
		Diversity	Land use	20.6	27.8	51.5	
			Physical structure	20.9	21.4	57.7	
			Economics	25	31.1	43.9	
		Density		20.4	24.5	55.1	
		Parking		10.9	19.2	69	

## 3 Material and method

### 3.1 Study site location

**Fig. 4:**
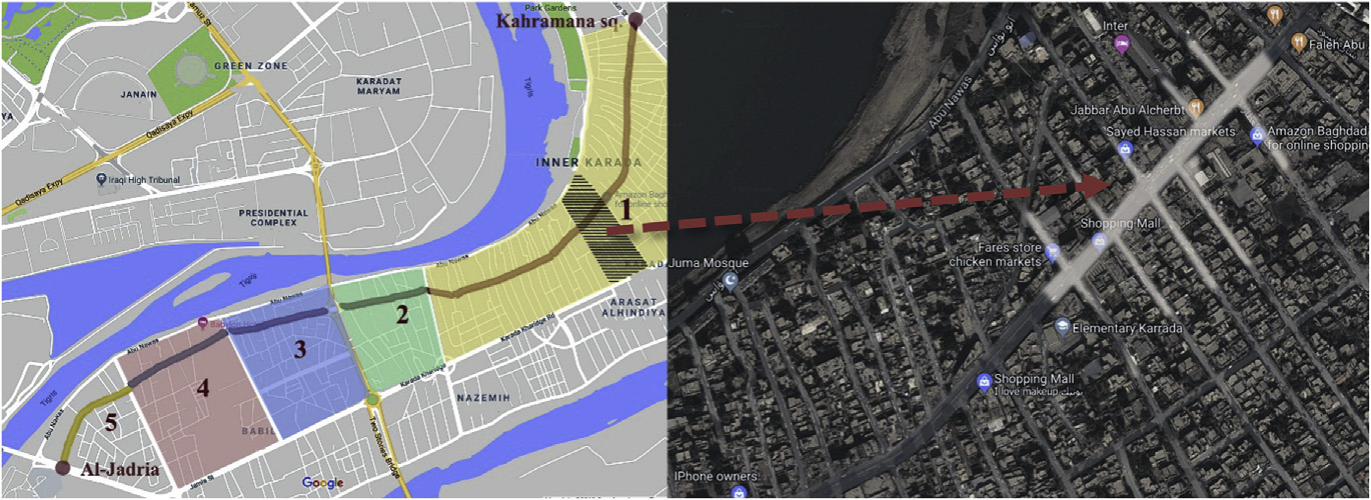
Five intersections divide Al-Karada inner street into five main mixed-use zones. Source: Adopted from [50], [51] and modified by the author.

**Fig. 5:**
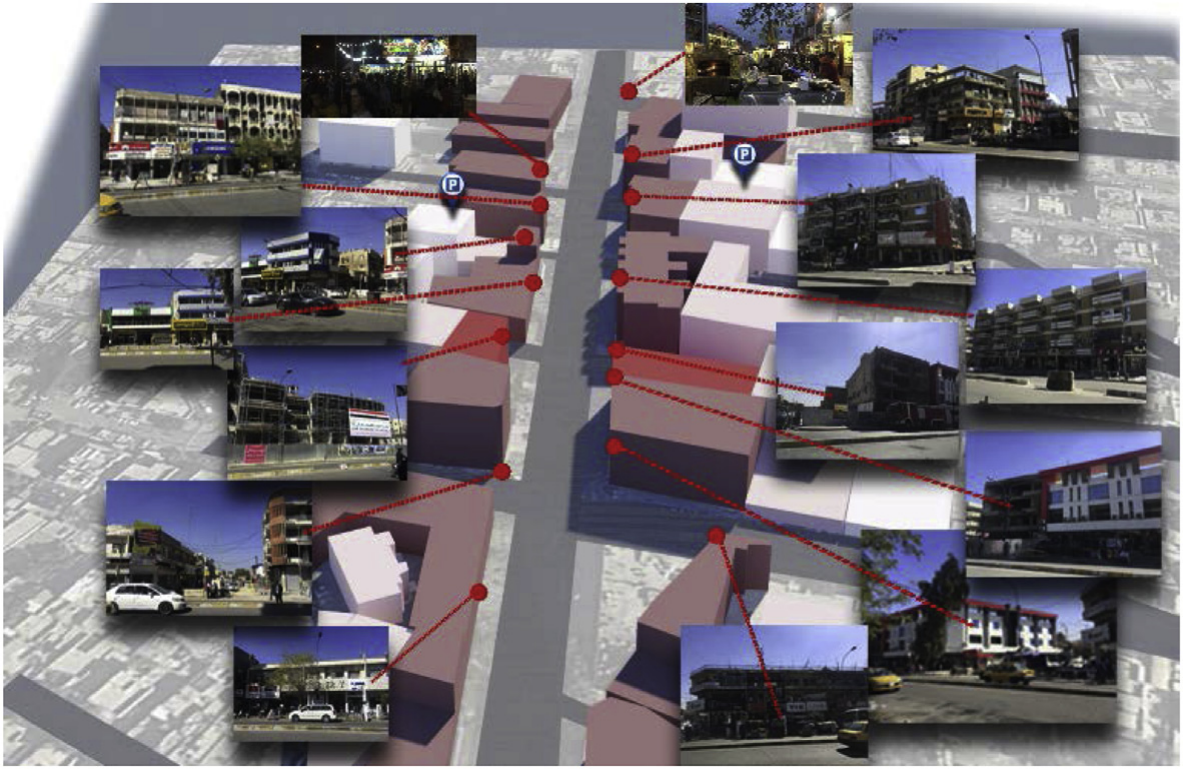
Study area. Source: captured and created by author.

Baghdad (33.33° N, 44.43° E) is the capital of Iraq, with an extremely hot summer and somewhat rainy, cold weather in winter. Al-Karada district is one of Baghdad's most famous neighbourhoods, located on a peninsula on the eastern side of the Tigris River (the Al-Rusafa side of Baghdad), surrounded by the Tigris on three sides (‘Baghdad’, n.d.). It has two sides along the city, one called Al-Karada inner area and the other called Al-Karada outer area. The main street in Al-Karada inner area is a very lively commercial street with total length of 6.3 km (‘Google Earth Pro’, 2018). Each side of Al-Karada inner street has five sections, most with similar characteristics. Areas 1, 2, 3, and 4 are mixed-use commercial areas dominated by grocery shops and fish and barbecue kiosks; Area 5 is a residential area (Fig. 4) (see Fig. 5).

Area 1, is the longest, with numerous markets and shops. At the entrance are clothing stores, followed by grocery and fish markets with barbecue kiosks, then an area to sell and display paintings, and finally clothing stores and a goldsmith area. A 300-m section of Area 1 was chosen as the case study site. The site is a commercial area with many mixed multi-story buildings, in which shops occupy the ground floor and the first to third floors house apartments, medical clinics, offices, and storage. The area also includes mosques, a post office, and many other activities [52]. The study site was chosen for many reasons. First, it was in this area that a terrorist attack occurred on 3 July 2016, killing many residents and shop owners and resulting in loss of one of the most important elements of the street's vitality, safety. As a result, the street was abandoned for many months, until the residential area launched the ‘#Al-Karada-chooses-life’ campaign to revive the street. Since that time, the number of daily visitors has been 25%–30% lower than before the attack, according to shop owners and residents of the region. Another reason for selection of this site is that most properties in this area are privately owned. The individual behaviour of independent owners causes chaos in the street, which negatively affects its commercial function. So, Applying the developed liveability checklist will stimulate commercial activities which leads to increase the land value, provides opportunities for more jobs and mostly to offer welfare for all categories of street users (elderly, children, youth and people with special needs). As a result, Al-Karrada Inner Street has been selected to test the liveability checklist due to its strategic location in the heart of Baghdad city, its economic importance, and the location' potentialities which can increase the vitality of the street and improve its functional performance efficiency as well.

In addition to descriptively describing and analysing the concept of urban liveability for upgrading streets based on previous literature and theories to build a comprehensive framework in the form of an applicable checklist, this study examined the proposed checklist through a questionnaire as an analytical study of the existing conditions of the Al-Karada case study site, thereby indicating the most important local indicators for enhancing liveability and tested the validity of the variance answers. First of all, the questionnaire included two study samples. The sample included (100) person, (25) of them were urban design specialists and (75) person chosen randomly including street visitors of all ages, shop owners and residents. A questionnaire was prepared (Appendix 1), and anyone can answer it, however part of it was allocated to the specialized sample represented by urban designers and some of the samples couldn't answer them, while the rest of the survey paper were answered by both (the specialized and the general). Interviews were about (10–20 minutes). The response of the specialized sample was 100%, while the general response was about 85%. Some of them were conservative and abstained from interviewed or answering the questionnaire for feeling uncertainty and unsafety. Implementation of the questionnaire began on 4 November 2018, from 5:00 pm until 10:00 pm, and continued for four days at the peak hours for the presence of people on the street. Additionally, the same questionnaire was implemented through an electronic Google form is directed to experts in urban planning and design. The results explained in each aspect with the necessary charts of the total (100) participants' outcomes. Secondly, and for the validation of the questionnaire to the interrelated indicators and questions, a one-way analysis (ANOVA) is used to measure the significance of the liveable commercial streets checklist, taking into consideration the means of the population sample variance. Although the standard Dev. is a little bit high for the three opinions, it shows a significant result with f = 10.35 at p < 0.05 (Appendix 2) [53]. Thus, the questionnaire is valid, and the checklist can be used to test the streets liveability.

### 3.2 Field measurement: survey campaign questionnaire

In addition to descriptively describing and analysing the concept of urban liveability for upgrading streets based on previous literature and theories to build a comprehensive framework in the form of an applicable checklist, this study examined the proposed checklist through a questionnaire and analytical study of the existing conditions of the Al-Karada case study site, thereby indicating the most important local indicators for enhancing liveability. The questionnaire included two study samples. First was a general sample, directed to the community and the shop owners, and second was a specialized sample. Implementation of the questionnaire for the first sample began on 4 November 2018, from 5:00 pm until 10:00 pm, and continued for four days at the peak hours for presence of people on the street. The questionnaire for the second sample was implemented through an electronic Google form directed with different questions to experts in urban planning and design and could not be answered by participants of the first sample. The results explained in each aspect with the necessary charts of the total (100) participants’ outcomes.

## 4 Results & discussion

### 4.1 Design and location aspect

**Fig. 6:**
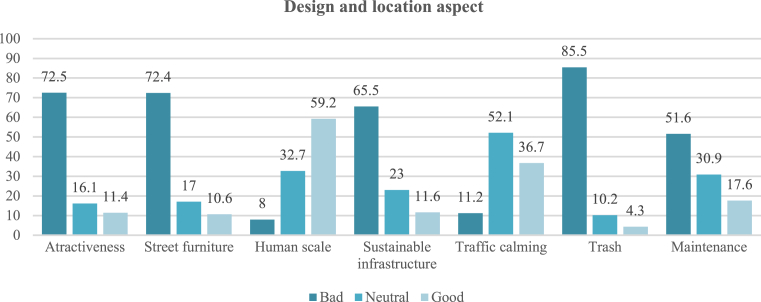
Results of rating for the design and location aspect show that five main indicators received a negative rating from more than 60% of participants. Only two indicators received an overall positive rating.

The design and location aspect include the street's characteristics and physical elements, which contribute to changing people's activities from necessary to optional, thus causing people to linger in the street. This aspect includes four main indicators: design components, natural components, street location components, and in-between space components. The design components include attractiveness (art; unique details; accessibility), street furniture (number of public and commercial seats; lighting; signs and instructions), human scale (distance and biometrics; edge segments; proportion of building height to street width), sustainable infrastructure (porous pavement; street water management; renewable energies; pedestrian and bicycle paths), traffic mitigation, trash management, and maintenance. The results of the questionnaire showed significant differences in rating between these categories. Trash management received the most negative rating (85.5% of participants), with strong criticism for lack of trash containers and visible trash accumulation; this was followed by negative ratings for attractiveness (72.5%) and street furniture (72.4%). Sustainable infrastructure received a negative rating from 65.5% of participants for inability to accommodate the street's increasing needs. Street maintenance was rated negatively by 61.6% for absence of cleaning and repair services. In contrast, human scale and traffic mitigation received positive evaluations from 59.2% and 36.7% of participants, respectively. This was due to the width of the street and sidewalk, which are considered relatively small and cosy, and the distance between components, which is close enough to achieve the human scale, as agreed upon by theorists. Two-way street traffic and a semi-shared vehicle pathway played a key role in mitigating traffic. All rating results are shown in Fig. 6.

**Fig. 7:**
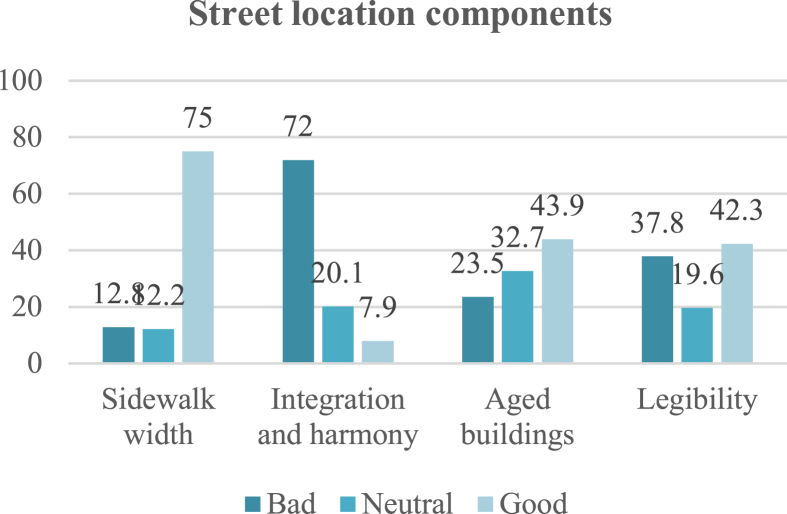
Rating of street location components. The integration and harmony sub-indicator received the highest negative rating.

Natural components include topography, trees, plants, weather, and pollution level. Protection from the elements received the highest negative rating (74%) on the questionnaire due to scarcity of trees and shade. This was followed by trees and plants, with a 64.3% negative rating due to decrease in their number and the continuous need for maintenance and respacing. These factors led to increase in the level of noise and air pollution, which received a 61.3% negative rating, as shown in Fig. 7.

**Fig. 8:**
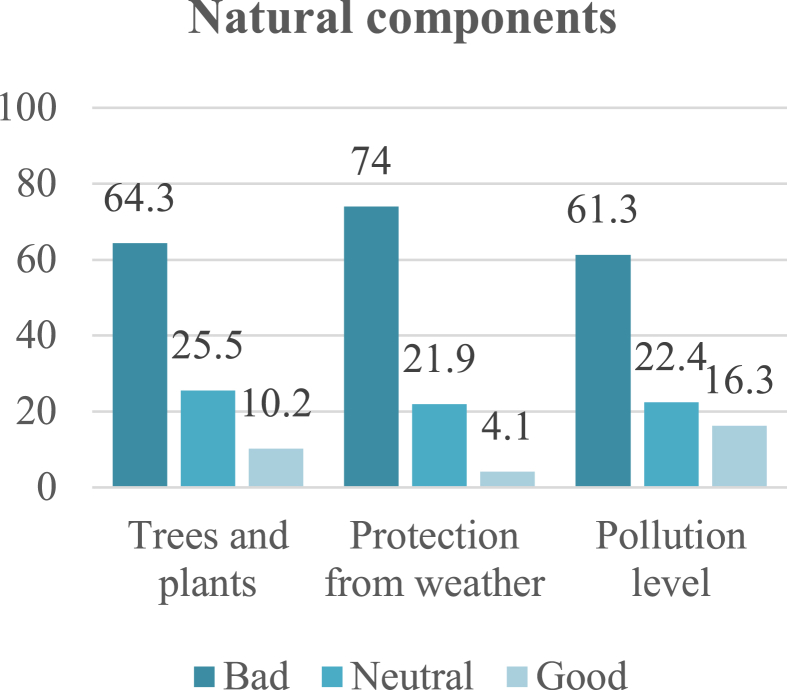
Rating of natural components. All sub-indicators received a more than 60% negative rating.

Street location components include sidewalk width, integration and harmony, aged buildings, distinction, and complexity. On one hand, sidewalk width received the highest majority positive rating (75%) among the specialized sample, followed by distinction (42.3%). On the other hand, aged buildings was rated most negatively (76.2%). Integration and harmony, referring to the integration of buildings with each other and the harmony of buildings with those adjacent in terms of height, materials, colour, size, window openings, or other details (except where differences are intended by the designer or imposed on the existing context) was rated negatively by 72% due to chaos and confusion (Fig. 8).

**Fig. 9:**
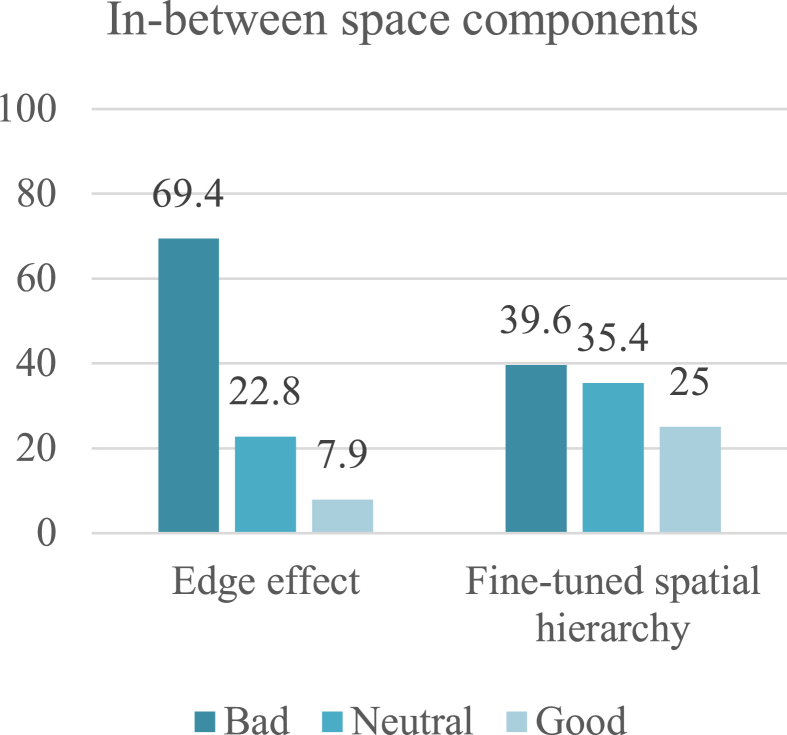
Rating of in-between space components. The edges effect received a negative rating from about 70% of participants, whereas fine-tuned spatial hierarchy received a negative rating from about 40%.

The final indicator in the design and location aspect is in-between space components, which comprise fine-tuned spatial hierarchy, enclosure, and the edges effect. The edges effect was rated most negatively (69.4%) due to the interference of spaces with street activities and the confusion of edges and their permeability. For the same reasons, fine-tuned spatial hierarchy received a 39.6% negative rating; the facades are transparent and permeable, but the boundaries between spaces and activities are confused and crowded, making it difficult to recognize or feel familiar towards them (Fig. 9).

### 4.2 Social and cultural aspect

Social and cultural indicators are resulted in a society's values, manners, and culture, which have a great role in social interaction and communication in the street. The aspect is divided into two main sections. First is social components, including vending and kiosks, personalization, shows and performances, activities, and needs and desires (participation, communication, food, people's presence, and variety of goods). Vending and kiosks received the most negative rating (72.6% of questionnaire participants). Few performances and shows are presented in the area, resulting in a majority negative rating of 76.2%. However, the activities available on the street are diverse, such as eating, shopping, walking, and more. Thus, the diversity of activities was rated positively by most participants (84.6%). Variety of goods was also rated positively (84.8%). Thus, these indicators show that the needs of a wide range of people of different ages and cultures are met, and they are attracted to stay in the area longer. The final social component is human needs and desires in the street, represented by participation, people's presence, food, communication, and variety of goods. People's presence, food, and variety of goods received high positive ratings, but communication and participation are difficult in the street because of the noise and crowds, resulting in negative ratings. The overall rating of human needs and desires was positive, at 65.5%.

**Fig. 10:**
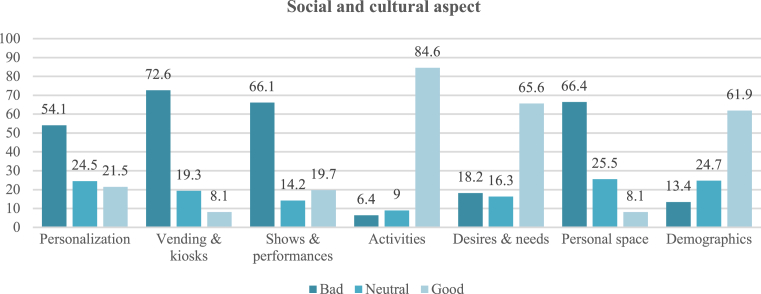
Ratings for the indicators of the social and cultural aspect.

The second main section of the social and cultural aspect is cultural components, which include sense of place, the age of the street (time), cultural factors, personal space, and demographics. Personal space index and demographics were measured by the questionnaire in this study; the other indicators were not, because they require sophisticated study of the behavioural structure of individuals. Evaluation of personal space availability on the street was negative among 66.4% of participants. This is due to the inability of street users to communicate or to stop and interact, affecting the length of their presence on the street and thus its vitality. Demographic diversity on the street was rated positively by 61.9%, showing that this indicator increases interaction between people and the diversity of their activities. Due to failure in some of the indicators, as described above, the street is in poor condition and requires some improvements to enhance its performance. Fig. 10 which shows the range of ratings for all measured indicators of the social and cultural aspect.

### 4.3 Urban planning aspect

The urban planning aspect is summarized by five main indicators: accessibility, context, diversity, parking and density. Accessibility includes the sub-indicators of connectivity, short blocks, effective transportation, multiple transportation options. Connectivity was calculated by the equation (connectivity ratio = number of paths/number of nodes) [54]. The output of the account is 1.2, and the ratio required for pedestrian community is 1.4; the length of the sectors was mostly within the required length or 153–183 m [54]. Thus, the area has sufficient connectivity for accessibility, enhancing its vitality. However, the bicycle route and footpath (effective transportation) are too narrow, and do not provide a comfortable pedestrian path. In addition, there are no bicycle paths, leading to a positive effective transportation rating of only 8.3%. The final factor in achieving accessibility is multiple transportation options, including public transportation, taxis, and private vehicles; 56.1% of participants rated this factor positively, indicating that the street suits a wide range of people's needs. Context involves the preservation of natural elements and urban centres. Because there is no specific advantage or extension of natural elements, this factor was neglected. As for urban centres, the street is itself considered one of the most important commercial streets of the city and an extension of the city centre; preservation of urban centres and places of symbolic value thus received a positive rating of 59.1%.

Diversity is one of the most important indicators of liveability, including land uses, physical components, and the economic level of the population. Land use in the study site varies between commercial, residential, religious, and health uses; its diversity was rated positively by 51.5% of the sample. The physical and economic components were evaluated positively by 57.7% and 43.9%, respectively. This diversity increases interaction between people and leads to variation in their activities and stimulation between them.

**Fig. 11:**
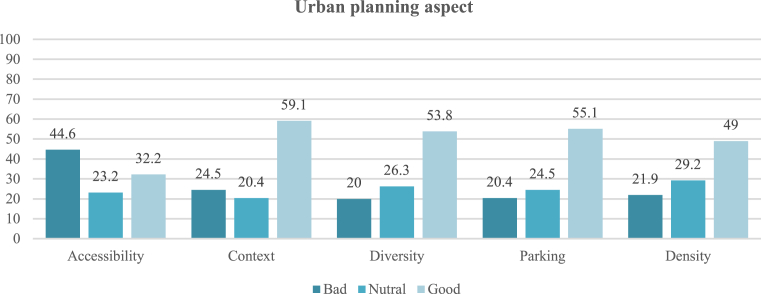
Rating of urban planning aspect indicators on the street. All indicators received a positive rating except accessibility, although the negative rating was less than 60%.

Four car parks are available in the area, which can accommodate 240 cars, but they must be organized and designed according to the appropriate standards. Car parks are found in the secondary streets also; thus, the rating of this indicator was 55.1%. Finally, the density indicator was assessed positively by 69%, with a high density considered positive in terms of liveability because it leads to closeness among people and activities. These results show that the biggest problem for the street is accessibility; however, even this indicator's negative rating was not particularly high, as shown in Fig. 11, which presents the ratings for all urban planning aspect indicators on the street.

### 4.4 Summary of indicators for Al- Karada inner street

**Fig. 12:**
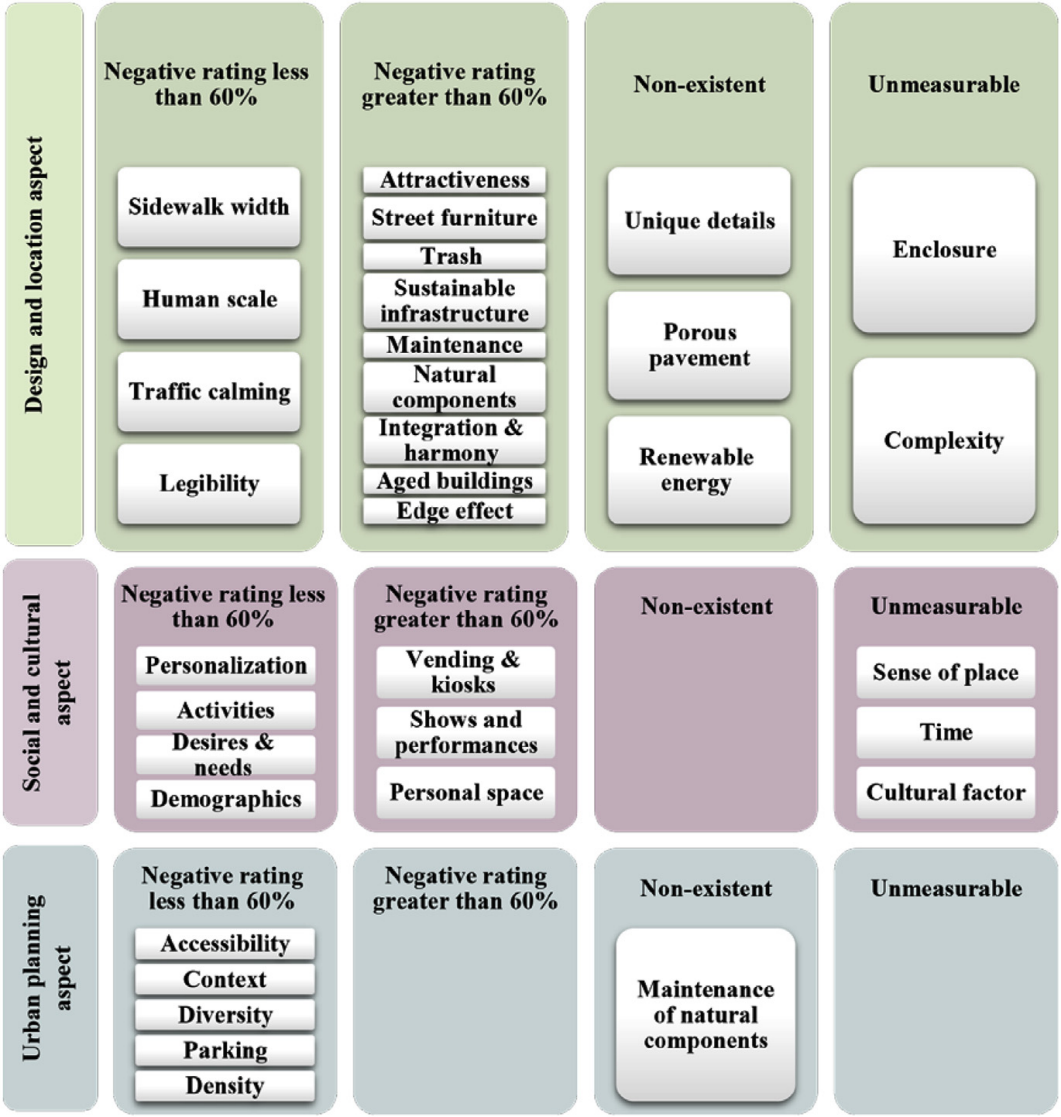
Summary of the three main aspect of commercial street liveability for Al-Karada inner street.

Indicator ratings were divided into four levels: indicators with a positive rating of less than 60%, indicators with a negative rating of more than 60%, indicators not measured because they require specialized study, and non-existent indicators (Fig. 12). The checklist developed in this study was able to identify the strength and weaknesses of Al-Karada inner street regarding the three key aspects of street liveability.

First, and as seen in Fig. 12, the urban planning aspect demonstrates the most efficient performance; all its sub-indicators could be measured and received a negative rating of less than 60%. In contrast, the design and location aspect demonstrate the most deficient performance, having many unmeasurable indicators and receiving the highest negative rating. The social and cultural aspect falls between the two, because people's perspectives and views are affected by street performance, which shows positive results on the urban level, but the street still struggles at the level of detailed design.

## 5 Conclusion

A theoretical, inductive review conducted by this research provided an extensive study of the most important theories and literature addressing the concept of liveability as an essential factor in upgrading commercial streets, creating a comprehensive knowledge base for the most important indicators to raise the efficiency of a street's performance and to increase its liveability. Based on this study, a checklist was developed evaluated, including the most important indicators of the design and location, social and cultural, and urban planning aspects. The list is the first of its kind, which combines those three aspects of liveability. The checklist was applied to an important liveable site, Al-Karada inner street, which lost its important attractive factors due to extraordinary external circumstances. This checklist provided a comprehensive information base that identified the street's most important strengths in liveability as well as its weaknesses and failures, opened the prospects to find solutions for increasing the liveability of Al-Karada inner Street and contribute to upgrading it. In the future, the checklist could be applied to multiple streets, allowing it to be more comprehensive. The more application of the checklist, the more indicators found. Eventually, the checklist represents a starting point to develop a future sophisticated list to measure the liveability in different street types.

## Declarations

### Author contribution statement

Noor Mazin Ghazi: Conceived and designed the experiments; Performed the experiments; Analyzed and interpreted the data; Contributed reagents, materials, analysis tools or data; Wrote the paper.

Zaynab R. Abaas: Analyzed and interpreted the data; Contributed reagents, materials, analysis tools or data; Wrote the paper.

### Funding statement

This research did not receive any specific grant from funding agencies in the public, commercial, or not-for-profit sectors.

### Competing interest statement

The authors declare no conflict of interest.

### Additional information

No additional information is available for this paper.
