# Supplementary material for: Toward liveable commercial streets: A case study of Al-Karada inner street in Baghdad
Source: Heliyon. 2019 May 20;5(5):e01652. doi: 10.1016/j.heliyon.2019.e01652 (PMC6529690; doi:10.1016/j.heliyon.2019.e01652)
Supplement: Appendix 1 [file mmc1.docx]

Appendix 1 **The questionnaire of the liveability in the commercial streets - Prepared by the author**

| (If you can’t answer some questions leave them to the specialized) | | | 1. while you are walking, do you feel that the street is | | |
| --- | --- | --- | --- | --- | --- |
| 1. How do you rate the edges segments number? | | | long | not long | short |
| few | acceptable | many | 1. You feel like your home boundary is   (answer this question only if you live in Al-Karada inner street) | | |
| 1. How do you rate street water management? | | | your house door | your building door | the whole street |
| bad | acceptable | good | 1. While you are walking in the street, how do you rate your ability to see the activities inside buildings? | | |
| 1. How do you rate sidewalk width? | | | i can’t see | i can hardly see | i can see well |
| narrow | acceptable | wide | 1. How do you rate commercial extension on the sidewalk? | | |
| 1. How do you rate harmony and integration between street components? | | | bad | acceptable | good |
| bad | acceptable | good | 1. How do you rate vending and kiosks condition? | | |
| 1. How do you rate aged buildings? | | | bad | acceptable | good |
| bad | acceptable | good | 1. Are there shows and performance on the street? | | |
| 1. How do you rate soft edges? | | | no |  | yes |
| bad | acceptable | good | 1. If yes, how do you rate it? | | |
| 1. How do you rate the maintenance of urban centers in the street? | | | bad | acceptable | good |
| bad | acceptable | good | 1. How do you rate the activities diversity? | | |
| 1. How do you rate the land uses mixing? | | | bad | acceptable | good |
| bad | acceptable | good | 1. How do you rate the ability to participate in events in the street? | | |
| 1. How do you rate the variety of economic levels? | | | bad | acceptable | good |
| bad | acceptable | good | 1. How do you rate people density on the street? | | |
| 1. How do you rate art & unique details on the street? | | | bad | acceptable | good |
| bad | acceptable | good | 1. How do you rate cafes and restaurants in the street? | | |
| 1. How do you rate the lighting in the street? | | | bad | acceptable | good |
| bad | acceptable | good | 1. Can you communicate in the street? If not or hardly, why? | | |
| 1. How do you rate the signs and instruction in the street? | | | i can’t | i can hardly | i can’t |
| bad | acceptable | good | 1. How do you rate the variety of goods? | | |
| 1. How do you rate the distances between people, goods and activates? | | | bad | acceptable | good |
| bad | acceptable | good | 1. The space you have on the street enough to feel | | |
| 1. How do you rate the walking paths? | | | uncomfortable | acceptable | comfortable |
| comfortable & attractive | acceptable | hard to walk | 1. How do you rate the transportation in the street? | | |
| 1. Cars in the street are | | | bad | acceptable | good |
| slow | not fast | fast | 1. How do you rate the variety of transportation options? | | |
| 1. How do you rate trash handling? | | | few | acceptable | many |
| bad | acceptable | good | 1. How do you rate the physical structure of the street? | | |
| 1. How do you rate maintenance in the street? | | | bad | acceptable | good |
| bad | acceptable | good | 1. How do you rate the visitors and activities concentration in the street? | | |
| 1. How do you rate trees and planets condition? | | | bad | acceptable | good |
| bad | acceptable | good | 1. How do you rate the parking availability? | | |
| 1. How do you rate shading and protection areas? | | | bad | acceptable | good |
| bad | acceptable | good | 1. How do you rate accessibility? | | |
| 1. How do you rate seating, eating, resting area? | | | bad | acceptable | good |
| bad | acceptable | good |  | | |
