# Supplementary material for: Toward liveable commercial streets: A case study of Al-Karada inner street in Baghdad
Source: Heliyon. 2019 May 20;5(5):e01652. doi: 10.1016/j.heliyon.2019.e01652 (PMC6529690; doi:10.1016/j.heliyon.2019.e01652)

Appendix 2 **One-way analyses (ANOVA) is used to measure the significance of the livable commercial street’s checklist**
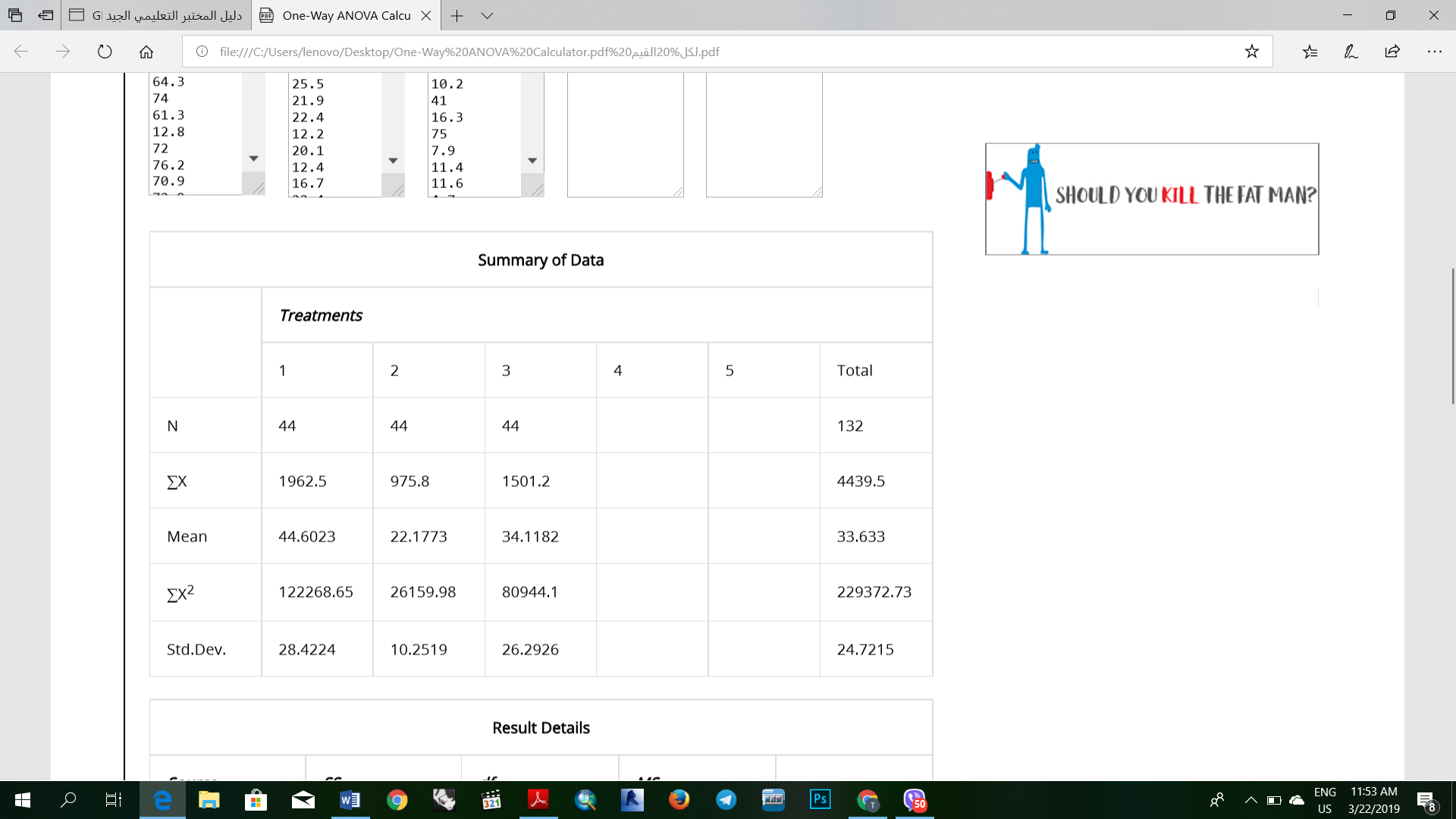


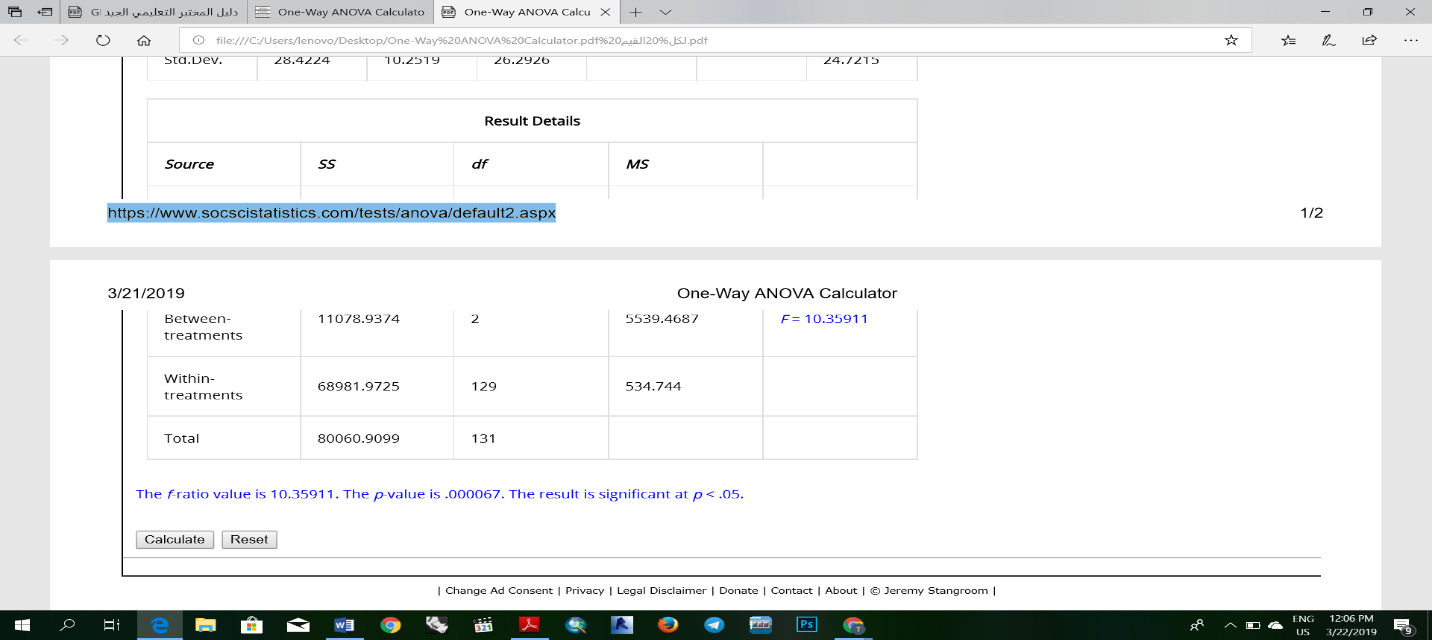

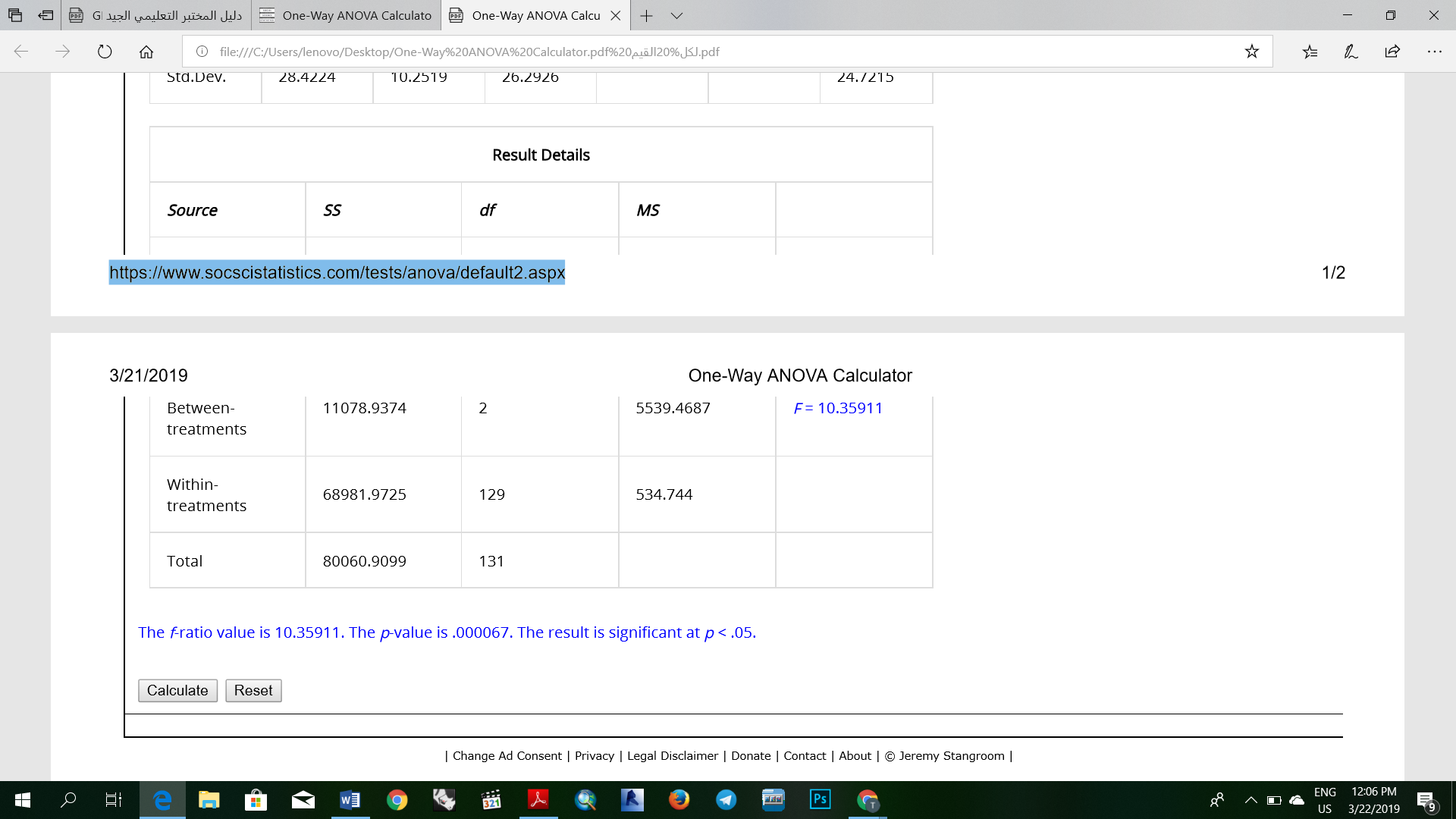

Supplement: Appendix 2 [file mmc2.docx]
